# Di-μ_3_-oxido-di-μ_2_-oxido-tetra­oxido­bis­(1,1,2,2-tetra­methyl­ethylenedicyclo­penta­dien­yl)­dimolyb­denum(IV)­dimolybdenum(VI) hexa­hydrate

**DOI:** 10.1107/S1600536808031668

**Published:** 2008-10-09

**Authors:** Takiya J. Ahmed, Lev N. Zakharov, David R. Tyler

**Affiliations:** a Department of Chemistry, 1253 University of Oregon, Eugene, Oregon 97403-1253, USA

## Abstract

The title compound, [Mo_4_(C_16_H_20_)_2_O_8_]·6H_2_O, is a centrosymmetric *ansa*-molybdocene complex in which two dinuclear [C_2_Me_4_(η^5^-C_5_H_4_)_2_]Mo(μ_2_-O)_2_MoO_2_ units dimerize by forming two μ_3_-O bridges between three Mo atoms. The *ansa*-molybdocene [C_2_Me_4_(η^5^-C_5_H_4_)_2_]Mo unit has a typical bent-sandwich metallocene structure with an inter-ring angle of 127.98 (8)°. The Mo atom in the bridging (μ_2_-O)(μ_3_-O)_2_MoO_2_ group has a distorted trigonal–bipyramidal coordination. The Mo—(μ_3_-O) and Mo—(μ_2_-O) bond distances inside the units [2.0869 (14) and 2.1014 (15) Å, respectively] are slightly longer than the Mo(−*x* + 1, −*y* + 1, −*z*)—(μ_3_-O) bond distance between the units [1.9986 (14) Å]. The solvent water mol­ecules together with complex O atoms form a network of O—H⋯O hydrogen bonds.

## Related literature

For related structures, see: Prout & Daran (1978 ▶); Adam & Green (1981 ▶); Daran & Prout (1977 ▶); Prout *et al.* (1974 ▶). For general synthesis and reactivity information on related tetramethylethylene-bridged ansa-molybdocene complexes, see: Ahmed *et al.* (2007 ▶).
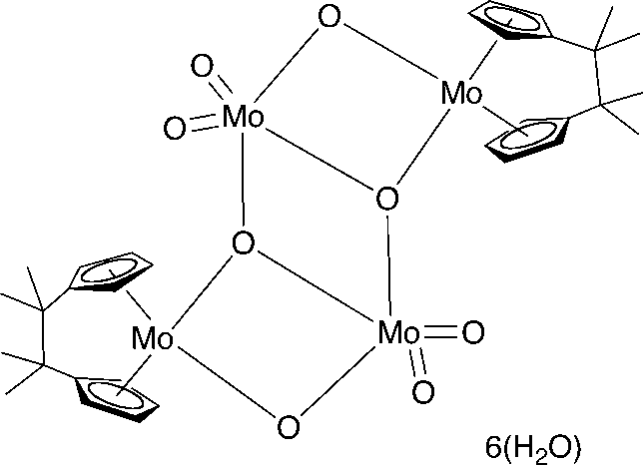


## Experimental

### 

#### Crystal data

[Mo_4_(C_16_H_20_)_2_O_8_]·6H_2_O
*M* *_r_* = 1044.50
Triclinic, 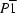

*a* = 7.4106 (7) Å
*b* = 9.3518 (8) Å
*c* = 13.5735 (12) Å
α = 93.365 (1)°
β = 98.604 (1)°
γ = 103.175 (1)°
*V* = 901.39 (14) Å^3^
*Z* = 1
Mo *K*α radiation
μ = 1.43 mm^−1^
*T* = 173 (2) K
0.30 × 0.09 × 0.07 mm

#### Data collection

Bruker APEX CCD diffractometer
Absorption correction: multi-scan (*SADABS*; Bruker, 2000 ▶) *T*_min_ = 0.673, *T*_max_ = 0.907
10175 measured reflections
3916 independent reflections
3621 reflections with *I* > 2σ(*I*)
*R*_int_ = 0.014

#### Refinement

*R*[*F* ^2^ > 2σ(*F* ^2^)] = 0.021
*wR*(*F* ^2^) = 0.054
*S* = 1.09
3916 reflections
250 parameters
H atoms treated by a mixture of independent and constrained refinement
Δρ_max_ = 0.46 e Å^−3^
Δρ_min_ = −0.41 e Å^−3^

### 

Data collection: *SMART* (Bruker, 2000 ▶); cell refinement: *SAINT* (Bruker, 2000 ▶); data reduction: *SAINT*; program(s) used to solve structure: *SHELXTL* (Sheldrick, 2008 ▶); program(s) used to refine structure: *SHELXTL*; molecular graphics: *SHELXTL*; software used to prepare material for publication: *SHELXTL*.

## Supplementary Material

Crystal structure: contains datablocks I, global. DOI: 10.1107/S1600536808031668/hb2806sup1.cif

Structure factors: contains datablocks I. DOI: 10.1107/S1600536808031668/hb2806Isup2.hkl

Additional supplementary materials:  crystallographic information; 3D view; checkCIF report

## Figures and Tables

**Table 1:** Selected bond lengths (Å)

Mo1—O1	2.0869 (14)
Mo1—O2	2.1014 (15)
Mo2—O4	1.7217 (16)
Mo2—O3	1.7237 (16)
Mo2—O2	1.8385 (15)
Mo2—O1^i^	1.9986 (14)
Mo2—O1	2.0481 (15)

Symmetry code: (i) 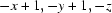.

**Table 2:** Hydrogen-bond geometry (Å, °)

*D*—H⋯*A*	*D*—H	H⋯*A*	*D*⋯*A*	*D*—H⋯*A*
O1*S*—H2*S*⋯O3*S*^ii^	0.76 (4)	2.02 (4)	2.771 (3)	169 (4)
O3*S*—H6*S*⋯O2*S*	0.80 (4)	1.99 (4)	2.792 (3)	175 (3)
O2*S*—H4*S*⋯O4^i^	0.76 (4)	2.16 (4)	2.908 (3)	167 (4)
O3*S*—H5*S*⋯O1*S*^iii^	0.81 (4)	2.06 (4)	2.850 (3)	163 (3)
O2*S*—H3*S*⋯O1*S*	0.76 (4)	2.12 (4)	2.865 (3)	165 (4)
O1*S*—H1*S*⋯O3^i^	0.82 (3)	2.01 (3)	2.816 (3)	167 (3)

Symmetry codes: (i) 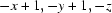; (ii) 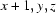; (iii) 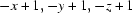.

**Fig. 1.**
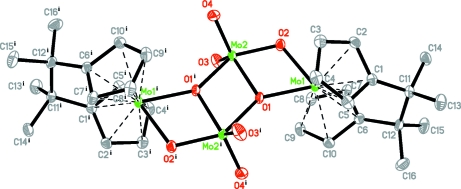
The structure of the [{C2Me4(η5-C5H4)2}Mo2O4]2 unit in (I) with 50% probability displacement ellipsoids [Symmetry code (i): 1-x,1-y,-z]. The H atoms were omitted for clarity.

**Fig. 2.**
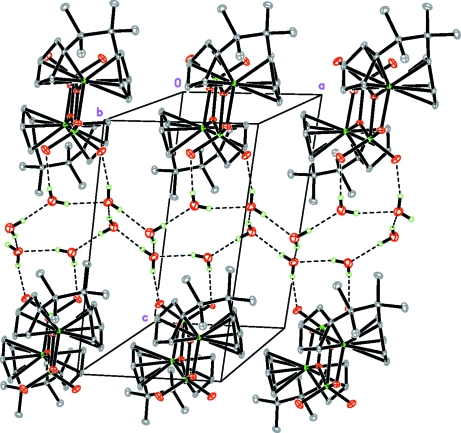
The network of H-bonds (dashed lines) in the structure of (I).

## supplementary crystallographic information

### Comment

We previously reported the synthesis and characterization of *ansa*-molybdocenes bridged by tetramethylethylene linkages (Ahmed *et al.*,  2007). Of those species, the monomeric [{C_2_Me_4_(η^5^-C_5_H_4_)_2_}Mo(OH)(OH_2_)][C_7_H_7_SO_3_] was exploited for catalytic hydration and hydrolysis reactions in aqueous solution.

The title complex, (I), was obtained as large needles from the slow oxidation of the [{C_2_Me_4_(η^5^-C_5_H_4_)_2_}Mo(OH)(OH_2_)][C_7_H_7_SO_3_] catalyst in the presence of 0.50 ml ethyl acetate in water at approximately pH 4. It is uncertain whether the title compound exists in equilibrium with the catalytic complex in water under anaerobic conditions. The compound is a centrosymmetric *ansa*-molybdocene complex in which two {C_2_Me_4_(η^5^-C_5_H_4_)_2_}Mo(µ_2_-O)_2_MoO_2_ units are connected by two µ_3_-O bridges between three Mo atoms (Fig. 1). The *ansa*-molybdocene {C_2_Me_4_(η^5^-C_5_H_4_)_2_}Mo_2_ has a typical bent-sandwich structure with an angle of 127.98 (8)° between the average planes of the C_5_-rings. The Mo atom in the bridging (µ_2_-0)(µ_3_-O)_2_MoO_2_ group has a distorted trigonal bipyramidal coordination. The Mo(1)-(µ_3_-O) and Mo(1)-(µ_2_-O) bond distances, 2.0869 (14) and 2.1014 (15) Å, respectively, are slightly longer than the O(1)-Mo(2)(1-*x*, 1-*y*,-*z*) bond distance, 1.9986 (14) Å (Table 1). Solvent water molecules together with O atoms of the (µ_2_-0)(µ_3_-O)_2_MoO_2_ groups form a network of H-bonds (Table 2, Fig. 2).

Analogous non-*ansa* oxides, [(η^5^-C_5_H_4_R)_2_Mo_2_O_4_]_2_ (where R = H or Me), were reported in previous literature (Prout *et al.*, 1974, Prout & Daran, 1978, Adam & Green, 1981). In those cases, the tetranuclear molybdenum dimers were prepared by reaction of the respective molybdocene dichloride, (η^5^-C_5_H_4_R)_2_MoCl_2_, and sodium molybdate. The structure of the [(η^5^-C_5_H_4_Me)_2_Mo_2_O_4_]_2_ was determined by single crystal diffraction methods (Prout & Daran, 1978). The central framework in this compound is similar to that found in the title compound.

### Experimental

Ethyl acetate (7 ml) was added to an NMR tube containing [{C_2_Me_4_(η^5^5-C_5_H_4_)_2_}Mo(OH)(OH_2_)][C_7_H_7_SO_3_] (0.0023 mg, 0.0026 mmol) in 0.50 ml D_2_O.  The mixture was allowed to react over two months at 323 K during which time the ethyl acetate was converted to ethanol and acetic acid.  As the reaction proceeded, the mixture turned dark in color and crystalline needles of (I) suitable for X-ray analysis appeared on the NMR tube walls.

### Refinement

The positions of the H atoms in solvent water molecules were found from the residual density and their positions were freely refined with isotropic thermal parameters. Other H atoms were positioned geometrically and refined in a rigid group model, C—H = 0.98 Å (C_5_-rings) and 0.96 Å (Me-groups); *U*_iso_(H) = 1.2 and 1.5 *U*_eq_(C), respectively.

### Crystal data

**Table:**

[Mo_4_(C_16_H_20_)_2_O_8_]·6H_2_O	*Z* = 1
*M**_r_* = 1044.50	*F*(000) = 524
Triclinic, *P*1	*D*_x_ = 1.924 Mg m^−^^3^
Hall symbol: -P 1	Mo *K*α radiation, λ = 0.71073 Å
*a* = 7.4106 (7) Å	Cell parameters from 6153 reflections
*b* = 9.3518 (8) Å	θ = 2.6–28.2°
*c* = 13.5735 (12) Å	µ = 1.43 mm^−^^1^
α = 93.365 (1)°	*T* = 173 K
β = 98.604 (1)°	Cut block, orange
γ = 103.175 (1)°	0.30 × 0.09 × 0.07 mm
*V* = 901.39 (14) Å^3^	

### Data collection

**Table:**

Bruker APEX CCD diffractometer	3916 independent reflections
Radiation source: fine-focus sealed tube	3621 reflections with *I* > 2σ(*I*)
graphite	*R*_int_ = 0.014
ω scans	θ_max_ = 27.0°, θ_min_ = 1.5°
Absorption correction: multi-scan (SADABS; Bruker, 2000)	*h* = −9→9
*T*_min_ = 0.673, *T*_max_ = 0.907	*k* = −11→11
10175 measured reflections	*l* = −17→17

### Refinement

**Table:**

Refinement on *F*^2^	Primary atom site location: structure-invariant direct methods
Least-squares matrix: full	Secondary atom site location: difference Fourier map
*R*[*F*^2^ > 2σ(*F*^2^)] = 0.021	Hydrogen site location: inferred from neighbouring sites
*wR*(*F*^2^) = 0.054	H atoms treated by a mixture of independent and constrained refinement
*S* = 1.09	*w* = 1/[σ^2^(*F*_o_^2^) + (0.0277*P*)^2^ + 0.6752*P*] where *P* = (*F*_o_^2^ + 2*F*_c_^2^)/3
3916 reflections	(Δ/σ)_max_ = 0.001
250 parameters	Δρ_max_ = 0.46 e Å^−^^3^
0 restraints	Δρ_min_ = −0.41 e Å^−^^3^

### Special details

**Table:**

Geometry. All esds (except the esd in the dihedral angle between two l.s. planes) are estimated using the full covariance matrix. The cell esds are taken into account individually in the estimation of esds in distances, angles and torsion angles; correlations between esds in cell parameters are only used when they are defined by crystal symmetry. An approximate (isotropic) treatment of cell esds is used for estimating esds involving l.s. planes.
Refinement. Refinement of F^2^ against ALL reflections. The weighted R-factor wR and goodness of fit S are based on F^2^, conventional R-factors R are based on F, with F set to zero for negative F^2^. The threshold expression of F^2^ > 2sigma(F^2^) is used only for calculating R-factors(gt) etc. and is not relevant to the choice of reflections for refinement. R-factors based on F^2^ are statistically about twice as large as those based on F, and R- factors based on ALL data will be even larger.

### Fractional atomic coordinates and isotropic or equivalent isotropic displacement parameters (Å^2^)

**Table:**

	*x*	*y*	*z*	*U*_iso_*/*U*_eq_	
Mo1	0.29716 (2)	0.241010 (19)	0.116363 (13)	0.01106 (6)	
Mo2	0.40229 (2)	0.365828 (19)	−0.091484 (13)	0.01260 (6)	
O1	0.4426 (2)	0.42724 (16)	0.05904 (11)	0.0143 (3)	
O2	0.2879 (2)	0.19918 (16)	−0.03835 (11)	0.0177 (3)	
O3	0.2214 (2)	0.40535 (18)	−0.17230 (13)	0.0252 (4)	
O4	0.5547 (3)	0.30518 (18)	−0.15855 (14)	0.0267 (4)	
C1	0.3632 (3)	0.0962 (2)	0.23884 (16)	0.0145 (4)	
C2	0.3911 (3)	0.0317 (2)	0.14559 (16)	0.0164 (4)	
H2A	0.3272	−0.0696	0.1132	0.020*	
C3	0.5455 (3)	0.1302 (3)	0.11548 (17)	0.0184 (5)	
H3B	0.6036	0.1113	0.0556	0.022*	
C4	0.6129 (3)	0.2517 (3)	0.18767 (17)	0.0181 (5)	
H4B	0.7234	0.3356	0.1862	0.022*	
C5	0.5003 (3)	0.2357 (2)	0.26298 (17)	0.0171 (4)	
H5A	0.5205	0.3032	0.3256	0.021*	
C6	0.0704 (3)	0.2045 (2)	0.21417 (16)	0.0149 (4)	
C7	−0.0141 (3)	0.1375 (2)	0.11433 (16)	0.0156 (4)	
H7A	−0.0799	0.0315	0.0951	0.019*	
C8	−0.0106 (3)	0.2551 (3)	0.05167 (17)	0.0180 (5)	
H8A	−0.0662	0.2437	−0.0210	0.022*	
C9	0.0778 (3)	0.3893 (3)	0.10838 (18)	0.0196 (5)	
H9A	0.0953	0.4889	0.0829	0.024*	
C10	0.1328 (3)	0.3613 (2)	0.20837 (17)	0.0172 (4)	
H10A	0.1881	0.4376	0.2663	0.021*	
C11	0.2217 (3)	0.0245 (2)	0.30248 (16)	0.0162 (4)	
C12	0.0833 (3)	0.1277 (2)	0.31082 (16)	0.0171 (4)	
C13	0.3320 (4)	0.0042 (3)	0.40383 (18)	0.0244 (5)	
H13A	0.4152	−0.0610	0.3933	0.037*	
H13B	0.2439	−0.0397	0.4471	0.037*	
H13C	0.4073	0.1003	0.4357	0.037*	
C14	0.1182 (3)	−0.1308 (2)	0.25306 (18)	0.0208 (5)	
H14A	0.2091	−0.1916	0.2493	0.031*	
H14B	0.0551	−0.1234	0.1854	0.031*	
H14C	0.0248	−0.1763	0.2930	0.031*	
C15	−0.1156 (3)	0.0416 (3)	0.3224 (2)	0.0258 (5)	
H15A	−0.1963	0.1106	0.3272	0.039*	
H15B	−0.1079	−0.0098	0.3831	0.039*	
H15C	−0.1688	−0.0306	0.2640	0.039*	
C16	0.1514 (4)	0.2459 (3)	0.40115 (18)	0.0265 (5)	
H16A	0.0605	0.3070	0.4029	0.040*	
H16B	0.2741	0.3081	0.3948	0.040*	
H16C	0.1630	0.1975	0.4631	0.040*	
O1S	0.8478 (3)	0.5646 (2)	0.37887 (16)	0.0327 (4)	
O2S	0.4926 (4)	0.6405 (3)	0.36850 (19)	0.0472 (6)	
O3S	0.2122 (3)	0.6526 (2)	0.48301 (17)	0.0336 (4)	
H1S	0.843 (4)	0.569 (3)	0.319 (3)	0.032 (9)*	
H2S	0.951 (6)	0.596 (4)	0.402 (3)	0.051 (12)*	
H3S	0.592 (6)	0.631 (5)	0.380 (3)	0.063 (14)*	
H4S	0.483 (5)	0.668 (4)	0.317 (3)	0.047 (11)*	
H5S	0.219 (5)	0.596 (4)	0.526 (3)	0.038 (9)*	
H6S	0.296 (5)	0.646 (4)	0.453 (3)	0.041 (10)*	

### Atomic displacement parameters (Å^2^)

**Table:**

	*U*^11^	*U*^22^	*U*^33^	*U*^12^	*U*^13^	*U*^23^
Mo1	0.00936 (9)	0.01184 (10)	0.01173 (10)	0.00140 (7)	0.00206 (7)	0.00287 (7)
Mo2	0.01369 (10)	0.01183 (10)	0.01116 (10)	0.00041 (7)	0.00266 (7)	0.00113 (7)
O1	0.0162 (8)	0.0134 (7)	0.0109 (7)	−0.0019 (6)	0.0029 (6)	0.0023 (6)
O2	0.0212 (8)	0.0147 (8)	0.0137 (7)	−0.0026 (6)	0.0032 (6)	0.0012 (6)
O3	0.0246 (9)	0.0191 (8)	0.0254 (9)	−0.0005 (7)	−0.0079 (7)	0.0040 (7)
O4	0.0319 (10)	0.0184 (8)	0.0336 (10)	0.0051 (7)	0.0195 (8)	0.0007 (7)
C1	0.0126 (10)	0.0155 (10)	0.0162 (10)	0.0058 (8)	0.0002 (8)	0.0048 (8)
C2	0.0162 (11)	0.0137 (10)	0.0205 (11)	0.0055 (8)	0.0033 (9)	0.0030 (8)
C3	0.0128 (10)	0.0220 (11)	0.0229 (12)	0.0073 (9)	0.0048 (9)	0.0055 (9)
C4	0.0097 (10)	0.0200 (11)	0.0251 (12)	0.0050 (8)	0.0002 (8)	0.0067 (9)
C5	0.0135 (10)	0.0182 (11)	0.0186 (11)	0.0044 (8)	−0.0023 (8)	0.0032 (8)
C6	0.0113 (10)	0.0169 (11)	0.0185 (11)	0.0049 (8)	0.0058 (8)	0.0037 (8)
C7	0.0090 (10)	0.0208 (11)	0.0176 (11)	0.0030 (8)	0.0034 (8)	0.0052 (9)
C8	0.0098 (10)	0.0252 (12)	0.0201 (11)	0.0053 (9)	0.0013 (8)	0.0084 (9)
C9	0.0144 (11)	0.0204 (11)	0.0286 (13)	0.0089 (9)	0.0071 (9)	0.0111 (9)
C10	0.0139 (10)	0.0160 (11)	0.0231 (12)	0.0051 (8)	0.0055 (9)	0.0022 (9)
C11	0.0165 (10)	0.0175 (11)	0.0150 (10)	0.0039 (8)	0.0031 (8)	0.0058 (8)
C12	0.0200 (11)	0.0195 (11)	0.0141 (10)	0.0060 (9)	0.0062 (8)	0.0055 (8)
C13	0.0262 (13)	0.0291 (13)	0.0189 (12)	0.0083 (10)	0.0011 (10)	0.0098 (10)
C14	0.0210 (12)	0.0158 (11)	0.0251 (12)	0.0010 (9)	0.0063 (9)	0.0052 (9)
C15	0.0217 (12)	0.0334 (14)	0.0283 (13)	0.0103 (10)	0.0125 (10)	0.0153 (11)
C16	0.0353 (14)	0.0311 (14)	0.0162 (11)	0.0124 (11)	0.0072 (10)	0.0015 (10)
O1S	0.0298 (12)	0.0441 (12)	0.0231 (11)	0.0051 (9)	0.0032 (9)	0.0115 (9)
O2S	0.0370 (14)	0.0829 (19)	0.0293 (12)	0.0235 (13)	0.0123 (10)	0.0135 (12)
O3S	0.0328 (11)	0.0361 (11)	0.0341 (11)	0.0074 (9)	0.0109 (9)	0.0124 (9)

### Geometric parameters (Å, °)

**Table:**

Mo1—O1	2.0869 (14)	C7—C8	1.428 (3)
Mo1—O2	2.1014 (15)	C7—H7A	1.0000
Mo1—C2	2.259 (2)	C8—C9	1.399 (3)
Mo1—C6	2.272 (2)	C8—H8A	1.0000
Mo1—C1	2.272 (2)	C9—C10	1.415 (3)
Mo1—C7	2.284 (2)	C9—H9A	1.0000
Mo1—C10	2.286 (2)	C10—H10A	1.0000
Mo1—C3	2.310 (2)	C11—C13	1.537 (3)
Mo1—C5	2.319 (2)	C11—C14	1.540 (3)
Mo1—C8	2.354 (2)	C11—C12	1.572 (3)
Mo1—C9	2.360 (2)	C12—C16	1.543 (3)
Mo1—C4	2.372 (2)	C12—C15	1.545 (3)
Mo2—O4	1.7217 (16)	C13—H13A	0.9800
Mo2—O3	1.7237 (16)	C13—H13B	0.9800
Mo2—O2	1.8385 (15)	C13—H13C	0.9800
Mo2—O1^i^	1.9986 (14)	C14—H14A	0.9800
Mo2—O1	2.0481 (15)	C14—H14B	0.9800
O1—Mo2^i^	1.9985 (14)	C14—H14C	0.9800
C1—C2	1.433 (3)	C15—H15A	0.9800
C1—C5	1.445 (3)	C15—H15B	0.9800
C1—C11	1.521 (3)	C15—H15C	0.9800
C2—C3	1.424 (3)	C16—H16A	0.9800
C2—H2A	1.0000	C16—H16B	0.9800
C3—C4	1.402 (3)	C16—H16C	0.9800
C3—H3B	1.0000	O1S—H1S	0.82 (3)
C4—C5	1.407 (3)	O1S—H2S	0.76 (4)
C4—H4B	1.0000	O2S—H3S	0.76 (4)
C5—H5A	1.0000	O2S—H4S	0.76 (4)
C6—C10	1.444 (3)	O3S—H5S	0.81 (4)
C6—C7	1.445 (3)	O3S—H6S	0.80 (4)
C6—C12	1.533 (3)		
			
O1—Mo1—O2	69.58 (6)	C1—C2—H2A	126.2
O1—Mo1—C2	127.58 (7)	Mo1—C2—H2A	126.2
O2—Mo1—C2	91.12 (7)	C4—C3—C2	109.1 (2)
O1—Mo1—C6	133.26 (7)	C4—C3—Mo1	75.01 (13)
O2—Mo1—C6	132.42 (7)	C2—C3—Mo1	69.91 (12)
C2—Mo1—C6	96.13 (8)	C4—C3—H3B	125.3
O1—Mo1—C1	136.34 (7)	C2—C3—H3B	125.3
O2—Mo1—C1	127.87 (7)	Mo1—C3—H3B	125.3
C2—Mo1—C1	36.88 (8)	C3—C4—C5	108.7 (2)
C6—Mo1—C1	69.27 (8)	C3—C4—Mo1	70.18 (12)
O1—Mo1—C7	133.87 (7)	C5—C4—Mo1	70.51 (12)
O2—Mo1—C7	95.63 (7)	C3—C4—H4B	125.6
C2—Mo1—C7	94.87 (8)	C5—C4—H4B	125.6
C6—Mo1—C7	36.99 (8)	Mo1—C4—H4B	125.6
C1—Mo1—C7	87.77 (8)	C4—C5—C1	107.6 (2)
O1—Mo1—C10	96.48 (7)	C4—C5—Mo1	74.60 (13)
O2—Mo1—C10	132.96 (7)	C1—C5—Mo1	69.88 (12)
C2—Mo1—C10	128.54 (8)	C4—C5—H5A	126.0
C6—Mo1—C10	36.95 (8)	C1—C5—H5A	126.0
C1—Mo1—C10	93.79 (8)	Mo1—C5—H5A	126.0
C7—Mo1—C10	61.30 (8)	C10—C6—C7	107.47 (19)
O1—Mo1—C3	91.40 (7)	C10—C6—C12	125.1 (2)
O2—Mo1—C3	79.28 (7)	C7—C6—C12	127.30 (19)
C2—Mo1—C3	36.30 (8)	C10—C6—Mo1	72.04 (12)
C6—Mo1—C3	128.90 (8)	C7—C6—Mo1	71.97 (12)
C1—Mo1—C3	60.16 (8)	C12—C6—Mo1	124.10 (14)
C7—Mo1—C3	129.81 (8)	C8—C7—C6	106.60 (19)
C10—Mo1—C3	147.47 (8)	C8—C7—Mo1	74.77 (12)
O1—Mo1—C5	101.32 (7)	C6—C7—Mo1	71.04 (12)
O2—Mo1—C5	137.56 (7)	C8—C7—H7A	126.4
C2—Mo1—C5	60.90 (8)	C6—C7—H7A	126.4
C6—Mo1—C5	84.85 (8)	Mo1—C7—H7A	126.4
C1—Mo1—C5	36.67 (8)	C9—C8—C7	109.4 (2)
C7—Mo1—C5	116.53 (8)	C9—C8—Mo1	72.97 (12)
C10—Mo1—C5	88.38 (8)	C7—C8—Mo1	69.42 (12)
C3—Mo1—C5	59.11 (8)	C9—C8—H8A	125.3
O1—Mo1—C8	98.21 (7)	C7—C8—H8A	125.3
O2—Mo1—C8	78.13 (7)	Mo1—C8—H8A	125.3
C2—Mo1—C8	125.79 (8)	C8—C9—C10	108.8 (2)
C6—Mo1—C8	59.69 (8)	C8—C9—Mo1	72.50 (13)
C1—Mo1—C8	123.14 (8)	C10—C9—Mo1	69.41 (12)
C7—Mo1—C8	35.81 (7)	C8—C9—H9A	125.6
C10—Mo1—C8	59.07 (8)	C10—C9—H9A	125.6
C3—Mo1—C8	150.51 (8)	Mo1—C9—H9A	125.6
C5—Mo1—C8	143.69 (8)	C9—C10—C6	107.6 (2)
O1—Mo1—C9	78.84 (7)	C9—C10—Mo1	75.18 (13)
O2—Mo1—C9	97.77 (7)	C6—C10—Mo1	71.01 (12)
C2—Mo1—C9	153.50 (8)	C9—C10—H10A	125.9
C6—Mo1—C9	59.71 (8)	C6—C10—H10A	125.9
C1—Mo1—C9	127.20 (8)	Mo1—C10—H10A	125.9
C7—Mo1—C9	59.55 (8)	C1—C11—C13	107.72 (18)
C10—Mo1—C9	35.41 (8)	C1—C11—C14	109.32 (18)
C3—Mo1—C9	170.20 (8)	C13—C11—C14	106.88 (19)
C5—Mo1—C9	121.75 (8)	C1—C11—C12	107.16 (17)
C8—Mo1—C9	34.53 (8)	C13—C11—C12	113.68 (19)
O1—Mo1—C4	78.20 (7)	C14—C11—C12	111.97 (18)
O2—Mo1—C4	104.43 (7)	C6—C12—C16	109.04 (19)
C2—Mo1—C4	59.57 (8)	C6—C12—C15	109.10 (18)
C6—Mo1—C4	119.70 (8)	C16—C12—C15	106.4 (2)
C1—Mo1—C4	59.39 (8)	C6—C12—C11	106.92 (17)
C7—Mo1—C4	147.17 (8)	C16—C12—C11	112.75 (19)
C10—Mo1—C4	116.66 (8)	C15—C12—C11	112.60 (19)
C3—Mo1—C4	34.82 (8)	C11—C13—H13A	109.5
C5—Mo1—C4	34.89 (8)	C11—C13—H13B	109.5
C8—Mo1—C4	174.32 (8)	H13A—C13—H13B	109.5
C9—Mo1—C4	139.82 (8)	C11—C13—H13C	109.5
O4—Mo2—O3	109.85 (9)	H13A—C13—H13C	109.5
O4—Mo2—O2	103.42 (8)	H13B—C13—H13C	109.5
O3—Mo2—O2	103.62 (8)	C11—C14—H14A	109.5
O4—Mo2—O1^i^	95.84 (7)	C11—C14—H14B	109.5
O3—Mo2—O1^i^	97.04 (7)	H14A—C14—H14B	109.5
O2—Mo2—O1^i^	144.73 (6)	C11—C14—H14C	109.5
O4—Mo2—O1	127.83 (8)	H14A—C14—H14C	109.5
O3—Mo2—O1	121.19 (8)	H14B—C14—H14C	109.5
O2—Mo2—O1	75.67 (6)	C12—C15—H15A	109.5
O1^i^—Mo2—O1	69.18 (7)	C12—C15—H15B	109.5
Mo2^i^—O1—Mo2	110.82 (7)	H15A—C15—H15B	109.5
Mo2^i^—O1—Mo1	145.59 (8)	C12—C15—H15C	109.5
Mo2—O1—Mo1	103.59 (6)	H15A—C15—H15C	109.5
Mo2—O2—Mo1	110.95 (7)	H15B—C15—H15C	109.5
C2—C1—C5	107.47 (19)	C12—C16—H16A	109.5
C2—C1—C11	125.40 (19)	C12—C16—H16B	109.5
C5—C1—C11	127.0 (2)	H16A—C16—H16B	109.5
C2—C1—Mo1	71.08 (12)	C12—C16—H16C	109.5
C5—C1—Mo1	73.45 (12)	H16A—C16—H16C	109.5
C11—C1—Mo1	123.64 (14)	H16B—C16—H16C	109.5
C3—C2—C1	106.98 (19)	H1S—O1S—H2S	105 (3)
C3—C2—Mo1	73.79 (13)	H3S—O2S—H4S	106 (4)
C1—C2—Mo1	72.03 (12)	H5S—O3S—H6S	105 (3)
C3—C2—H2A	126.2		
			
O4—Mo2—O1—Mo2^i^	80.94 (10)	O2—Mo1—C5—C1	−93.12 (14)
O3—Mo2—O1—Mo2^i^	−85.59 (9)	C2—Mo1—C5—C1	−38.26 (12)
O2—Mo2—O1—Mo2^i^	176.97 (9)	C6—Mo1—C5—C1	61.70 (13)
O1^i^—Mo2—O1—Mo2^i^	0.0	C7—Mo1—C5—C1	41.99 (15)
O4—Mo2—O1—Mo1	−99.53 (9)	C10—Mo1—C5—C1	98.55 (13)
O3—Mo2—O1—Mo1	93.93 (9)	C3—Mo1—C5—C1	−80.37 (14)
O2—Mo2—O1—Mo1	−3.51 (6)	C8—Mo1—C5—C1	73.63 (18)
O1^i^—Mo2—O1—Mo1	179.52 (12)	C9—Mo1—C5—C1	111.06 (13)
O2—Mo1—O1—Mo2^i^	−177.62 (16)	C4—Mo1—C5—C1	−115.83 (19)
C2—Mo1—O1—Mo2^i^	−102.69 (15)	O1—Mo1—C6—C10	−6.53 (17)
C6—Mo1—O1—Mo2^i^	52.73 (18)	O2—Mo1—C6—C10	−108.72 (13)
C1—Mo1—O1—Mo2^i^	−53.65 (19)	C2—Mo1—C6—C10	154.11 (13)
C7—Mo1—O1—Mo2^i^	104.65 (15)	C1—Mo1—C6—C10	128.38 (14)
C10—Mo1—O1—Mo2^i^	48.79 (15)	C7—Mo1—C6—C10	−115.94 (18)
C3—Mo1—O1—Mo2^i^	−99.60 (15)	C3—Mo1—C6—C10	136.84 (13)
C5—Mo1—O1—Mo2^i^	−40.86 (16)	C5—Mo1—C6—C10	94.17 (13)
C8—Mo1—O1—Mo2^i^	108.37 (15)	C8—Mo1—C6—C10	−77.68 (14)
C9—Mo1—O1—Mo2^i^	79.64 (15)	C9—Mo1—C6—C10	−37.48 (13)
C4—Mo1—O1—Mo2^i^	−67.17 (15)	C4—Mo1—C6—C10	95.80 (14)
O2—Mo1—O1—Mo2	3.17 (6)	O1—Mo1—C6—C7	109.41 (13)
C2—Mo1—O1—Mo2	78.09 (10)	O2—Mo1—C6—C7	7.22 (16)
C6—Mo1—O1—Mo2	−126.48 (9)	C2—Mo1—C6—C7	−89.95 (13)
C1—Mo1—O1—Mo2	127.14 (9)	C1—Mo1—C6—C7	−115.69 (14)
C7—Mo1—O1—Mo2	−74.56 (11)	C10—Mo1—C6—C7	115.94 (18)
C10—Mo1—O1—Mo2	−130.42 (8)	C3—Mo1—C6—C7	−107.22 (14)
C3—Mo1—O1—Mo2	81.19 (8)	C5—Mo1—C6—C7	−149.89 (13)
C5—Mo1—O1—Mo2	139.93 (8)	C8—Mo1—C6—C7	38.25 (12)
C8—Mo1—O1—Mo2	−70.84 (8)	C9—Mo1—C6—C7	78.46 (13)
C9—Mo1—O1—Mo2	−99.57 (8)	C4—Mo1—C6—C7	−148.27 (12)
C4—Mo1—O1—Mo2	113.62 (8)	O1—Mo1—C6—C12	−127.28 (16)
O4—Mo2—O2—Mo1	129.77 (9)	O2—Mo1—C6—C12	130.54 (16)
O3—Mo2—O2—Mo1	−115.59 (9)	C2—Mo1—C6—C12	33.37 (18)
O1^i^—Mo2—O2—Mo1	8.53 (16)	C1—Mo1—C6—C12	7.63 (17)
O1—Mo2—O2—Mo1	3.62 (7)	C7—Mo1—C6—C12	123.3 (2)
O1—Mo1—O2—Mo2	−3.68 (7)	C10—Mo1—C6—C12	−120.7 (2)
C2—Mo1—O2—Mo2	−133.74 (9)	C3—Mo1—C6—C12	16.1 (2)
C6—Mo1—O2—Mo2	126.90 (9)	C5—Mo1—C6—C12	−26.57 (18)
C1—Mo1—O2—Mo2	−137.18 (9)	C8—Mo1—C6—C12	161.6 (2)
C7—Mo1—O2—Mo2	131.26 (9)	C9—Mo1—C6—C12	−158.2 (2)
C10—Mo1—O2—Mo2	75.83 (12)	C4—Mo1—C6—C12	−24.9 (2)
C3—Mo1—O2—Mo2	−99.24 (9)	C10—C6—C7—C8	−3.2 (2)
C5—Mo1—O2—Mo2	−88.12 (12)	C12—C6—C7—C8	173.5 (2)
C8—Mo1—O2—Mo2	99.85 (9)	Mo1—C6—C7—C8	−66.90 (14)
C9—Mo1—O2—Mo2	71.29 (9)	C10—C6—C7—Mo1	63.74 (14)
C4—Mo1—O2—Mo2	−74.94 (9)	C12—C6—C7—Mo1	−119.5 (2)
O1—Mo1—C1—C2	−94.33 (14)	O1—Mo1—C7—C8	6.30 (17)
O2—Mo1—C1—C2	5.73 (16)	O2—Mo1—C7—C8	−60.66 (13)
C6—Mo1—C1—C2	134.00 (14)	C2—Mo1—C7—C8	−152.27 (14)
C7—Mo1—C1—C2	101.14 (13)	C6—Mo1—C7—C8	113.99 (19)
C10—Mo1—C1—C2	162.19 (13)	C1—Mo1—C7—C8	171.50 (14)
C3—Mo1—C1—C2	−38.41 (12)	C10—Mo1—C7—C8	75.95 (14)
C5—Mo1—C1—C2	−115.66 (18)	C3—Mo1—C7—C8	−141.40 (14)
C8—Mo1—C1—C2	107.07 (14)	C5—Mo1—C7—C8	147.93 (13)
C9—Mo1—C1—C2	149.37 (13)	C9—Mo1—C7—C8	35.06 (13)
C4—Mo1—C1—C2	−78.92 (13)	C4—Mo1—C7—C8	171.41 (14)
O1—Mo1—C1—C5	21.33 (17)	O1—Mo1—C7—C6	−107.69 (13)
O2—Mo1—C1—C5	121.39 (13)	O2—Mo1—C7—C6	−174.65 (12)
C2—Mo1—C1—C5	115.66 (18)	C2—Mo1—C7—C6	93.74 (13)
C6—Mo1—C1—C5	−110.34 (14)	C1—Mo1—C7—C6	57.51 (13)
C7—Mo1—C1—C5	−143.20 (13)	C10—Mo1—C7—C6	−38.04 (12)
C10—Mo1—C1—C5	−82.16 (13)	C3—Mo1—C7—C6	104.61 (14)
C3—Mo1—C1—C5	77.25 (14)	C5—Mo1—C7—C6	33.94 (15)
C8—Mo1—C1—C5	−137.27 (13)	C8—Mo1—C7—C6	−113.99 (19)
C9—Mo1—C1—C5	−94.98 (14)	C9—Mo1—C7—C6	−78.93 (13)
C4—Mo1—C1—C5	36.74 (12)	C4—Mo1—C7—C6	57.4 (2)
O1—Mo1—C1—C11	145.17 (15)	C6—C7—C8—C9	2.0 (2)
O2—Mo1—C1—C11	−114.77 (17)	Mo1—C7—C8—C9	−62.35 (16)
C2—Mo1—C1—C11	−120.5 (2)	C6—C7—C8—Mo1	64.37 (14)
C6—Mo1—C1—C11	13.50 (16)	O1—Mo1—C8—C9	−56.30 (14)
C7—Mo1—C1—C11	−19.36 (18)	O2—Mo1—C8—C9	−123.32 (14)
C10—Mo1—C1—C11	41.68 (18)	C2—Mo1—C8—C9	153.97 (13)
C3—Mo1—C1—C11	−158.9 (2)	C6—Mo1—C8—C9	79.57 (14)
C5—Mo1—C1—C11	123.8 (2)	C1—Mo1—C8—C9	108.95 (14)
C8—Mo1—C1—C11	−13.4 (2)	C7—Mo1—C8—C9	119.12 (19)
C9—Mo1—C1—C11	28.9 (2)	C10—Mo1—C8—C9	36.36 (13)
C4—Mo1—C1—C11	160.6 (2)	C3—Mo1—C8—C9	−164.06 (15)
C5—C1—C2—C3	1.1 (2)	C5—Mo1—C8—C9	65.78 (19)
C11—C1—C2—C3	−175.59 (19)	O1—Mo1—C8—C7	−175.42 (13)
Mo1—C1—C2—C3	66.06 (15)	O2—Mo1—C8—C7	117.57 (13)
C5—C1—C2—Mo1	−64.94 (14)	C2—Mo1—C8—C7	34.86 (17)
C11—C1—C2—Mo1	118.4 (2)	C6—Mo1—C8—C7	−39.55 (13)
O1—Mo1—C2—C3	5.24 (16)	C1—Mo1—C8—C7	−10.16 (17)
O2—Mo1—C2—C3	70.07 (13)	C10—Mo1—C8—C7	−82.75 (14)
C6—Mo1—C2—C3	−157.03 (13)	C3—Mo1—C8—C7	76.8 (2)
C1—Mo1—C2—C3	−114.45 (19)	C5—Mo1—C8—C7	−53.3 (2)
C7—Mo1—C2—C3	165.83 (14)	C9—Mo1—C8—C7	−119.12 (19)
C10—Mo1—C2—C3	−137.42 (13)	C7—C8—C9—C10	−0.1 (3)
C5—Mo1—C2—C3	−76.42 (14)	Mo1—C8—C9—C10	−60.21 (15)
C8—Mo1—C2—C3	146.22 (13)	C7—C8—C9—Mo1	60.14 (15)
C9—Mo1—C2—C3	−179.91 (17)	O1—Mo1—C9—C8	122.93 (14)
C4—Mo1—C2—C3	−36.04 (13)	O2—Mo1—C9—C8	55.62 (13)
O1—Mo1—C2—C1	119.69 (12)	C2—Mo1—C9—C8	−52.9 (2)
O2—Mo1—C2—C1	−175.47 (12)	C6—Mo1—C9—C8	−79.51 (14)
C6—Mo1—C2—C1	−42.58 (13)	C1—Mo1—C9—C8	−96.18 (15)
C7—Mo1—C2—C1	−79.72 (13)	C7—Mo1—C9—C8	−36.36 (13)
C10—Mo1—C2—C1	−22.97 (16)	C10—Mo1—C9—C8	−118.64 (19)
C3—Mo1—C2—C1	114.45 (19)	C5—Mo1—C9—C8	−140.58 (13)
C5—Mo1—C2—C1	38.03 (12)	C4—Mo1—C9—C8	179.09 (13)
C8—Mo1—C2—C1	−99.33 (14)	O1—Mo1—C9—C10	−118.43 (13)
C9—Mo1—C2—C1	−65.5 (2)	O2—Mo1—C9—C10	174.27 (13)
C4—Mo1—C2—C1	78.41 (13)	C2—Mo1—C9—C10	65.7 (2)
C1—C2—C3—C4	0.4 (2)	C6—Mo1—C9—C10	39.13 (13)
Mo1—C2—C3—C4	65.27 (16)	C1—Mo1—C9—C10	22.46 (17)
C1—C2—C3—Mo1	−64.87 (14)	C7—Mo1—C9—C10	82.28 (14)
O1—Mo1—C3—C4	66.83 (13)	C5—Mo1—C9—C10	−21.94 (16)
O2—Mo1—C3—C4	135.74 (14)	C8—Mo1—C9—C10	118.64 (19)
C2—Mo1—C3—C4	−117.32 (19)	C4—Mo1—C9—C10	−62.27 (18)
C6—Mo1—C3—C4	−87.41 (15)	C8—C9—C10—C6	−1.9 (2)
C1—Mo1—C3—C4	−78.28 (14)	Mo1—C9—C10—C6	−64.08 (14)
C7—Mo1—C3—C4	−135.84 (13)	C8—C9—C10—Mo1	62.15 (16)
C10—Mo1—C3—C4	−37.5 (2)	C7—C6—C10—C9	3.2 (2)
C5—Mo1—C3—C4	−35.54 (13)	C12—C6—C10—C9	−173.64 (19)
C8—Mo1—C3—C4	176.29 (14)	Mo1—C6—C10—C9	66.86 (15)
O1—Mo1—C3—C2	−175.85 (13)	C7—C6—C10—Mo1	−63.70 (14)
O2—Mo1—C3—C2	−106.93 (13)	C12—C6—C10—Mo1	119.5 (2)
C6—Mo1—C3—C2	29.91 (17)	O1—Mo1—C10—C9	60.26 (13)
C1—Mo1—C3—C2	39.04 (13)	O2—Mo1—C10—C9	−7.77 (17)
C7—Mo1—C3—C2	−18.52 (17)	C2—Mo1—C10—C9	−148.67 (13)
C10—Mo1—C3—C2	79.78 (19)	C6—Mo1—C10—C9	−114.96 (19)
C5—Mo1—C3—C2	81.79 (14)	C1—Mo1—C10—C9	−162.24 (13)
C8—Mo1—C3—C2	−66.4 (2)	C7—Mo1—C10—C9	−76.87 (14)
C4—Mo1—C3—C2	117.32 (19)	C3—Mo1—C10—C9	163.19 (15)
C2—C3—C4—C5	−1.8 (3)	C5—Mo1—C10—C9	161.47 (14)
Mo1—C3—C4—C5	60.21 (15)	C8—Mo1—C10—C9	−35.44 (13)
C2—C3—C4—Mo1	−62.02 (15)	C4—Mo1—C10—C9	140.28 (13)
O1—Mo1—C4—C3	−110.13 (14)	O1—Mo1—C10—C6	175.22 (12)
O2—Mo1—C4—C3	−45.07 (14)	O2—Mo1—C10—C6	107.18 (13)
C2—Mo1—C4—C3	37.59 (13)	C2—Mo1—C10—C6	−33.71 (16)
C6—Mo1—C4—C3	116.49 (14)	C1—Mo1—C10—C6	−47.29 (13)
C1—Mo1—C4—C3	80.68 (14)	C7—Mo1—C10—C6	38.08 (12)
C7—Mo1—C4—C3	80.78 (19)	C3—Mo1—C10—C6	−81.86 (19)
C10—Mo1—C4—C3	158.49 (13)	C5—Mo1—C10—C6	−83.57 (13)
C5—Mo1—C4—C3	119.3 (2)	C8—Mo1—C10—C6	79.51 (14)
C9—Mo1—C4—C3	−166.48 (13)	C9—Mo1—C10—C6	114.96 (19)
O1—Mo1—C4—C5	130.55 (14)	C4—Mo1—C10—C6	−104.76 (13)
O2—Mo1—C4—C5	−164.39 (12)	C2—C1—C11—C13	117.9 (2)
C2—Mo1—C4—C5	−81.73 (14)	C5—C1—C11—C13	−58.1 (3)
C6—Mo1—C4—C5	−2.83 (16)	Mo1—C1—C11—C13	−152.36 (16)
C1—Mo1—C4—C5	−38.64 (13)	C2—C1—C11—C14	2.1 (3)
C7—Mo1—C4—C5	−38.5 (2)	C5—C1—C11—C14	−173.9 (2)
C10—Mo1—C4—C5	39.17 (15)	Mo1—C1—C11—C14	91.9 (2)
C3—Mo1—C4—C5	−119.3 (2)	C2—C1—C11—C12	−119.4 (2)
C9—Mo1—C4—C5	74.20 (17)	C5—C1—C11—C12	64.5 (3)
C3—C4—C5—C1	2.5 (2)	Mo1—C1—C11—C12	−29.7 (2)
Mo1—C4—C5—C1	62.48 (14)	C10—C6—C12—C16	6.5 (3)
C3—C4—C5—Mo1	−60.01 (15)	C7—C6—C12—C16	−169.6 (2)
C2—C1—C5—C4	−2.2 (2)	Mo1—C6—C12—C16	97.7 (2)
C11—C1—C5—C4	174.4 (2)	C10—C6—C12—C15	122.3 (2)
Mo1—C1—C5—C4	−65.59 (15)	C7—C6—C12—C15	−53.8 (3)
C2—C1—C5—Mo1	63.38 (14)	Mo1—C6—C12—C15	−146.47 (17)
C11—C1—C5—Mo1	−120.0 (2)	C10—C6—C12—C11	−115.6 (2)
O1—Mo1—C5—C4	−49.33 (14)	C7—C6—C12—C11	68.2 (3)
O2—Mo1—C5—C4	22.71 (18)	Mo1—C6—C12—C11	−24.4 (2)
C2—Mo1—C5—C4	77.57 (14)	C1—C11—C12—C6	30.3 (2)
C6—Mo1—C5—C4	177.53 (14)	C13—C11—C12—C6	149.24 (19)
C1—Mo1—C5—C4	115.83 (19)	C14—C11—C12—C6	−89.5 (2)
C7—Mo1—C5—C4	157.82 (13)	C1—C11—C12—C16	−89.5 (2)
C10—Mo1—C5—C4	−145.62 (14)	C13—C11—C12—C16	29.4 (3)
C3—Mo1—C5—C4	35.46 (13)	C14—C11—C12—C16	150.66 (19)
C8—Mo1—C5—C4	−170.54 (13)	C1—C11—C12—C15	150.16 (19)
C9—Mo1—C5—C4	−133.11 (13)	C13—C11—C12—C15	−91.0 (2)
O1—Mo1—C5—C1	−165.16 (12)	C14—C11—C12—C15	30.3 (3)

Symmetry codes: (i) −*x*+1, −*y*+1, −*z*.

### Hydrogen-bond geometry (Å, °)

**Table:**

*D*—H···*A*	*D*—H	H···*A*	*D*···*A*	*D*—H···*A*
O1S—H2S···O3S^ii^	0.76 (4)	2.02 (4)	2.771 (3)	169 (4)
O3S—H6S···O2S	0.80 (4)	1.99 (4)	2.792 (3)	175 (3)
O2S—H4S···O4^i^	0.76 (4)	2.16 (4)	2.908 (3)	167 (4)
O3S—H5S···O1S^iii^	0.81 (4)	2.06 (4)	2.850 (3)	163 (3)
O2S—H3S···O1S	0.76 (4)	2.12 (4)	2.865 (3)	165 (4)
O1S—H1S···O3^i^	0.82 (3)	2.01 (3)	2.816 (3)	167 (3)

Symmetry codes: (ii) *x*+1, *y*, *z*; (i) −*x*+1, −*y*+1, −*z*; (iii) −*x*+1, −*y*+1, −*z*+1.
